# Computational quest for understanding the role of astrocyte signaling in synaptic transmission and plasticity

**DOI:** 10.3389/fncom.2012.00098

**Published:** 2012-12-21

**Authors:** Maurizio De Pittà, Vladislav Volman, Hugues Berry, Vladimir Parpura, Andrea Volterra, Eshel Ben-Jacob

**Affiliations:** ^1^School of Physics and Astronomy, Tel Aviv UniversityRamat Aviv, Israel; ^2^Center for Theoretical Biological Physics, University of California at San DiegoLa Jolla, CA, USA; ^3^Computational Neurobiology Laboratory, The Salk InstituteLa Jolla, CA, USA; ^4^L-3 Applied Technologies: Simulation, Engineering and TestingSan Diego, CA, USA; ^5^BEAGLE, INRIA Rhône-Alpes, Université de Lyon, LIRIS, UMR5205Villeurbanne, France; ^6^Department of Neurobiology, University of AlabamaBirmingham, AL, USA; ^7^Department of Biotechnology, University or RijekaRijeka, Croatia; ^8^Department of Basic Neurosciences, University of LausanneLausanne, Switzerland; ^9^Center for Theoretical Biological Physics, Rice UniversityHouston, TX, USA; ^10^Research and Development Unit, Assaf Harofeh Medical CenterZerifin, Israel

**Keywords:** astrocyte-synapse interactions, astrocyte modeling, calcium signaling, calcium encoding, gliotransmission, synaptic plasticity, metaplasticity, cortical maps

## Abstract

The complexity of the signaling network that underlies astrocyte-synapse interactions may seem discouraging when tackled from a theoretical perspective. Computational modeling is challenged by the fact that many details remain hitherto unknown and conventional approaches to describe synaptic function are unsuitable to explain experimental observations when astrocytic signaling is taken into account. Supported by experimental evidence is the possibility that astrocytes perform genuine information processing by means of their calcium signaling and are players in the physiological setting of the basal tone of synaptic transmission. Here we consider the plausibility of this scenario from a theoretical perspective, focusing on the modulation of synaptic release probability by the astrocyte and its implications on synaptic plasticity. The analysis of the signaling pathways underlying such modulation refines our notion of tripartite synapse and has profound implications on our understanding of brain function.

## Introduction

The simultaneous recognition that astrocytes sense neighboring neuronal activity and release neuroactive agents (or “gliotransmitters”) has been instrumental in the uncovering of the many roles played by these cells in the control of genesis, function and plasticity of synapses (Haydon, 2001; Ullian et al., 2004; Volterra and Meldolesi, 2005; Bains and Oliet, 2007; Santello and Volterra, 2009; Zorec et al., 2012). These findings initiated a conceptual revolution that leads to rethinking how brain communication works since they imply that information travels and is processed not just in the neuronal circuitry but in an expanded neuron-glial network (Haydon, 2001; Volterra and Meldolesi, 2005; Giaume et al., 2010). On the other hand the physiological need for astrocyte signaling in brain information processing and the modes of action of these cells in computational tasks remain largely undefined. This is due, to a large extent, both to the lack of conclusive experimental evidence, and to a substantial lack of a theoretical framework to address modeling and characterization of the many possible astrocyte functions. This review aims at introducing such a perspective providing a framework for future modeling efforts in the field based on preliminary theoretical studies on both astrocytic calcium signaling and gliotransmitter-mediated modulations of synaptic release probability.

## A theoretical framework for astrocyte-synapse interactions

**Figure 1:**
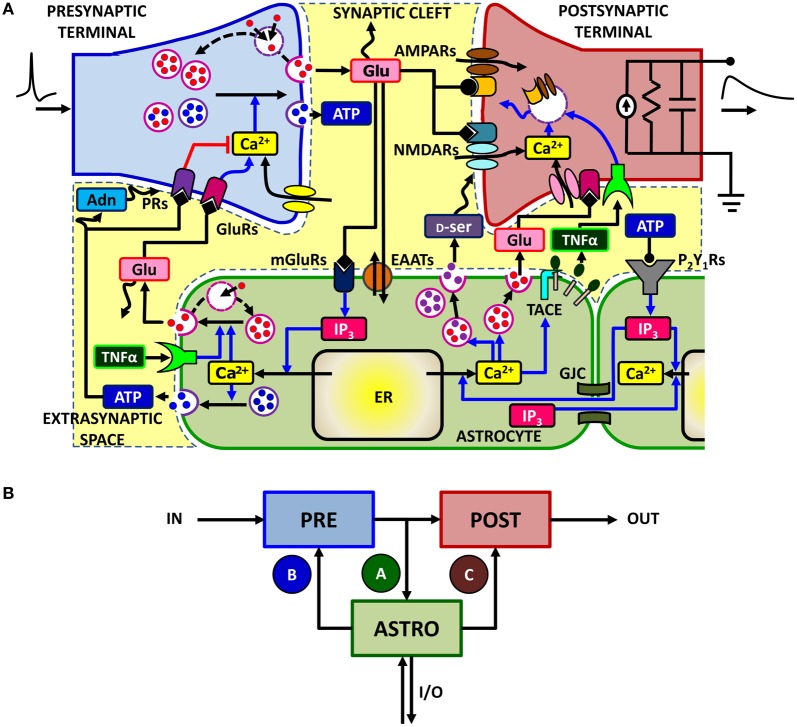
**The signaling network of astrocyte-synapse interactions. (A)** A simplified scheme of the different signaling pathways between synaptic terminals and astrocytes for the case of excitatory synapses in the hippocampus (see text for a detailed description). Action potentials arriving at the presynaptic terminal trigger release of glutamate, which can spill over from the synaptic cleft. Perisynaptic astrocytes take up glutamate using their plasma membrane transporters (EAATs) while glutamate, by acting on astrocytic metabotropic receptors (mGluRs), triggers Ca^2+^ signaling in the astrocyte. This signaling pathway includes production of IP_3_ and causes an increase of cytosolic Ca^2+^ due to efflux of this ion from the endoplasmic reticulum (ER). At some synapses, such as in the dentate gyrus, the same Ca^2+^ signaling pathway could also be mediated by astrocytic purinergic P_2_Y_1_ receptors, likely activated by synaptically-released ATP (see text for details). Astrocytic Ca^2+^ excitability can in turn lead to exocytotic release of several neuroactive substances (or “gliotransmitters”) such as glutamate (Glu), D-serine (D-ser) or ATP which can target specific receptors on pre- and post-synaptic terminals and differentially modulate synaptic transmission. Glutamate acting on presynaptic GluRs could enhance synaptic release, whereas ATP and its derivate adenosine (Adn) could depress it (*red path*) through presynaptic purinergic receptors (PRs). On the postsynaptic spines [depicted here by a standard RC circuit (Ermentrout and Terman, 2010)], the ensuing effect of gliotransmitters could substantially modify postsynaptic currents by enhancing activation of NMDA receptors (D-serine) or by altering expressions of AMPA receptors therein. Astrocytes could also release TNFα by Ca^2+^-dependent activation of the matrix metalloprotease TNFα-converting enzyme (TACE), while extracellular TNFα could in turn regulate glutamate release from the astrocyte as well as postsynaptic AMPAR expression. Moreover astrocytic Ca^2+^ could also propagate across different regions of the same cell or to other neighboring astrocytes by intracellular IP_3_ diffusion through gap junction channels (GJCs) or via extracellular ATP-dependent pathways, extending gliotransmission to some distal sites away from the considered synapse. For clarity both endocannabinoid-mediated Ca^2+^ signaling (Navarrete and Araque, 2008), retrograde activation of presynaptic glutamate receptors (Navarrete and Araque, 2010), regulation of postsynaptic NMDARs by presynaptic adenosine receptors (Deng et al., 2011), and the possibility for astrocyte-derived adenosine to enhance synaptic release (Panatier et al., 2011) are not included in this scheme. **(B)** Equivalent modeling scheme for astrocyte-synapse interactions. The astrocyte (ASTRO) constitutes a third active element of the tripartite synapse in addition to the presynaptic (PRE) and postsynaptic (POST) terminals. In its presence, the flow of input (IN) signals to the output (OUT) is no more unidirectional but presynaptically released neurotransmitter can affect astrocyte function through the interaction pathway *A*. In turn, the astrocyte can regulate both synaptic terminals via pathways *B* and *C*. In addition, the astrocyte could receive additional inputs from or send output to remote synapses in a heterosynaptic fashion (I/O).

Control of synaptic transmission and plasticity by astrocytes subtends a complex signaling network, which involves different biochemical pathways (Volterra and Meldolesi, 2005; Zorec et al., 2012). In general, synaptically-released neurotransmitter can spill out of the synaptic cleft and bind to metabotropic receptors found on the neighboring astrocytic processes triggering there inositol 1,4,5-trisphosphate (IP_3_)-mediated Ca^2+^ signaling. This was observed at both glutamatergic, cholinergic, noradrenergic, and GABAergic synapses in the hippocampus, in the thalamus and in the cortex (Volterra and Meldolesi, 2005; Haydon and Carmignoto, 2006; Santello and Volterra, 2009; Halassa and Haydon, 2010; Navarrete et al., 2012a,b). Figure 1A summarizes a number of observations made at the level of hippocampal glutamatergic synapses (Bains and Oliet, 2007; Santello and Volterra, 2009). There, synaptic glutamate can trigger Ca^2+^ signaling in the surrounding astrocytic processes via metabotropic glutamate receptors (mGluRs) (Pasti et al., 1997; Fiacco and McCarthy, 2004; Panatier et al., 2011). There is also evidence in the dentate gyrus that ATP, possibly synaptically-released, triggers astrocytic Ca^2+^ signaling through the activation of metabotropic purinergic P_2_Y_1_ receptors (Jourdain et al., 2007; Di Castro et al., 2011; Larsson et al., 2011; Santello et al., 2011). Following elevation of intracellular Ca^2+^, astrocytes can release glutamate as well as other chemical transmitters such as D-serine (D-ser) and ATP which can be converted into adenosine (Adn) in the extracellular milieu (Bezzi et al., 2004; Pascual et al., 2005; Montana et al., 2006; Henneberger et al., 2010; Parpura and Zorec, 2010). Astrocyte-released glutamate diffuses in the extrasynaptic space and may bind to glutamate receptors (GluRs), including mGluRs and NMDARs on neighboring presynaptic terminals, modulating the release of neurotransmitter (Fiacco and McCarthy, 2004; Jourdain et al., 2007; Perea and Araque, 2007; Bonansco et al., 2011; Di Castro et al., 2011). An analogous action on synaptic release could also be due to astrocyte-derived ATP and its derivative adenosine through presynaptic purinergic receptors (PRs), including both A_1_ and A_2_ receptors (Pascual et al., 2005; Halassa and Haydon, 2010; Panatier et al., 2011). On the postsynaptic side, astrocytic glutamate and D-serine may bind to extrasynaptic NR_2_B-containing and postsynaptic NMDARs respectively, modulating neuronal firing and participating in the induction of long-term potentiation (Fellin et al., 2004; Bains and Oliet, 2007; Henneberger et al., 2010). Astrocyte could also release tumor necrosis factor-α (TNFα) by Ca^2+^-dependent activation of TNFα-converting enzyme (TACE) (Bezzi et al., 2001; Santello and Volterra, 2012), which could strengthen excitatory synaptic transmission by promoting surface insertion of AMPA receptors (AMPARs) (Beattie et al., 2002; Stellwagen and Malenka, 2006; Bains and Oliet, 2007). This signaling route could also play a role in pathological states such as post-traumatic epilepsy (Balosso et al., 2009; Volman et al., 2011) or spinal cord injury (Stellwagen et al., 2005; Ferguson et al., 2008). On the other hand, extracellular levels of TNFα control glutamate release from astrocytes, ultimately modulating the astrocytic action on presynaptic function (Domercq et al., 2006; Santello et al., 2011). This intricate signaling network is further complicated by the possibility that astrocyte Ca^2+^ events are triggered by additional mechanisms, including the action of ATP released extracellularly by astrocytes themselves or of IP_3_ that diffuses intracellularly, from one astrocyte to another, through gap junction (GJCs) (Kang et al., 2005; Scemes and Giaume, 2006). At Schaffer collateral synapses, astrocytic Ca^2+^ increases could also be promoted by retrograde endocannabinoid signaling from postsynaptic terminals via activation of endocannabinoid CB_1_ receptors (Navarrete and Araque, 2008, 2010) (omitted from Figure 1A for clarity). Moreover, the relation between astrocytic Ca^2+^ and gliotransmitter release is not simple: some of the Ca^2+^ signals that can be generated in astrocytes are apparently not able to induce gliotransmitter release or its synaptic consequences (Fiacco et al., 2007; Agulhon et al., 2008, 2010; Petravicz et al., 2008; Lovatt et al., 2012), while gliotransmitters can also be released by mechanisms that are independent of Ca^2+^ signaling (Parpura and Zorec, 2010) (not included in Figure 1A for simplicity).

Despite its apparent complexity, the ensemble of astrocyte-synapse signaling interactions discussed above can be well captured by the modeling scheme of Figure 1B. This scheme shows the three essential components of astrocyte-regulated synapses, also referred to as “tripartite synapses” (Araque et al., 1999; Haydon, 2001): these are the pre- (PRE) and postsynaptic (POST) terminals, and the astrocyte, i.e., an astrocytic process surrounding the synaptic elements (ASTRO) (Araque et al., 1999; Haydon, 2001). Moreover, in addition to the classical neuronal path that leads from input presynaptic action potentials, commonly referred to as input spikes (IN), to the output postsynaptic current (OUT), further input and/or output pathways (I/O) could coexist due to the above-mentioned routes based on astrocytic Ca^2+^ signaling (Giaume et al., 2010).

Focusing on synapse-astrocyte coupling, three fundamental pathways are identified: one (**A**) from the synapse to the astrocyte whereby synaptically-released glutamate (or other synaptic agents) promotes Ca^2+^ signaling in the astrocyte and the other two (**B** and **C**) from the astrocyte to synaptic terminals, whereby glutamate or ATP released from the astrocyte affects synaptic function (Volterra and Meldolesi, 2005; Santello and Volterra, 2009). Additional pathways supported by other neuroactive agents such as D-serine or TNFα can also be evoked in parallel to those shown in Figure 1B but they would not alter the essence of the scheme. Moreover, although based on experimental results at excitatory synapses in the hippocampus, (Araque et al., 1998a,b; Fiacco and McCarthy, 2004; Jourdain et al., 2007; Perea and Araque, 2007; Andersson and Hanse, 2010; Santello et al., 2011), the modeling scheme in Figure 1B could also hold for other reported pathways such as GABA-evoked gliotransmission at interneuron-to-pyramidal cell synapses in the hippocampus (Kang et al., 1998; Serrano et al., 2006), or glia-mediated ATP release at hippocampal synapses (Pascual et al., 2005), in the hypothalamus (Gordon et al., 2009), and in the retina (Newman, 2003, 2005), or glial modulation of neuromuscular transmission (Robitaille, 1998; Rousse et al., 2010; Todd et al., 2010) (see Table 1 for a summary of the possible signaling pathways).

**Table 1:** **Transmitters, targeted receptors, and major effects on synaptic transmission by the signaling pathways A, B, C in Figure 1B (*in situ* and *in vivo* studies only)**.

**References**	**Sp.^1^**	**Prep.^2^**	**Area^3^**	**Syn. neurotr.^4^**	**Targeted receptor^5^**	**Gliotr.^6^**	**Targeted receptors^5^**		**Cell^7^**	**Effects^8^**
					**Pathway A**		**Pathway B**	**Pathway C**		
Wang et al., 2006	M	VV	BC	Glu	mGluR-I					
Porter and McCarthy, 1996	R	ST	CA1	Glu	mGluR, iGluR					
Perea and Araque, 2007	R	ST	CA1			Glu	mGluR-I		PY	↑synaptic release probability
Liu et al., 2004a,b	R	ST	CA1			Glu	KAR		IN	↓mIPSCs (frequency)
Liu et al., 2004a,b	R	ST	HIP			Glu	mGluR-II/III		IN	↑synaptic release probability
Bardoni et al., 2010	R	ST	DH			Glu		NMDAR	SGN	SICs; synchronous Ca^2+^ elevations in neighboring neurons
Kang et al., 2005	R	ST	CA1			Glu		iGluR	PY	SICs
Parri et al., 2001	R	ST	VBT			Glu		NMDAR	TCN	SICs
Bezzi et al., 1998	R	ST, VT	CA1, VC, COR			Glu		GluR	PY	Ca^2+^ elevations in neighboring neurons
Newman and Zahs, 1997	R	ST	Ret^m^			Glu^*^		iGluR	AC^*^	↑neuronal inhibition
Bonansco et al., 2011	R	ST	CA1			Glu	mGluR-I	NMDAR	PY	↑mEPSCs (frequency); SICs; control of t-LTP induction
Fiacco and McCarthy, 2004	M	ST	CA1	n.r.	n.r.	Glu	mGluR-I		PY	↑synaptic release probability
Pasti et al., 1997	R	ST	CA1, VC	Glu	mGluR	Glu^*^			PY	Ca^2+^ elevations in neighboring neurons
Pirttimaki et al., 2011	R	ST	VBT	n.r.	mGluR-I	Glu		NR_2_B-NMDAR	TCN	Long-term enhancement of SIC frequency
D'Ascenzo et al., 2007	M	ST	NAcc	Glu	mGluR_5_	Glu		NR_2_B-NMDAR	MSN	SICs, ↑neuronal firing
Fellin et al., 2004	R	ST	CA1	Glu	n.r.	Glu		NR_2_B-NMDAR	PY	SICs; synchronous Ca^2+^ elevations in neighboring neurons
Newman, 2005	R	ST	Ret^m^	ATP	n.r.					
Piet and Jahr, 2007	R	ST	CER^b^	Glu, ATP	AMPAR, P_2_YR					
Rieger et al., 2007	M	ST	OB	Glu, ATP	mGluR_1_, P_2_Y_1_R					
Beierlein and Regehr, 2006	R	ST	CER^b^	Glu, ATP	mGluR_1_, P_2_YR					
Newman, 2003	R	ST	Ret^m^			ATP		A_1_R	RN	↑K^+^ conductance; ↑neuronal inhibition
Torres et al., 2012	M	ST	CA1			ATP		P_2_Y_1_R	IN	↑neuronal firing
Schmitt et al., 2012	M	ST, VV	HIP	n.r.	n.r.	ATP	A_1_R		PY	↓fEPSP (slope)
Di Castro et al., 2011	R	ST	MLDG	ATP	P_2_Y_1_R	n.r.	n.r.		GC	↑synaptic release probability
Halassa et al., 2009	M	VV	COR	n.r.	n.r.	ATP	A_1_R		CN	↓fEPSP (slope)
Jourdain et al., 2007	R	ST	MLDG	ATP^*^	P_2_Y_1_R	Glu	NR_2_B-NMDARs		GC	↑synaptic release probability
Panatier et al., 2011	R	ST	CA1	Glu	mGluR_5_	ATP	A_2A_R		PY	↑synaptic release probability
Schipke et al., 2008	M	ST	BC	Glu	mGluR-I	ATP	A_1_R, P_2_YR		PY	↑neuronal inhibition; confinement of astrocytic Ca^2+^ signals
Pascual et al., 2005	M	VT, ST	CA1	Glu	n.r.	ATP	A_1_R		PY	↓fEPSP (slope); heterosynaptic depression; control of long-term plasticity (metaplasticity)
Zhang et al., 2003, 2004a	R	ST, VT	CA1	Glu	n.r.	ATP	A_1_R, P_2_YR		PY	↓EPSP (amplitude); heterosynaptic depression
Todd et al., 2010	F	VT	NMJ	n.r.	n.r.	ATP	A_1_R, A_2A_R		MF	PTD (A_1_R); PTP (A_2A_R)
Robitaille, 1998	F	VT	NMJ			n.r.	n.r.		MF	PTP; PTD
Bowser and Khakh, 2004	M	ST	CA1	ATP, Glu	P_2_Y_1_R, mGluR-I	ATP		P_2_Y_1_R	IN	SICs, ↑neuronal firing
Gordon et al., 2009	R	ST	PVN	Glu	mGluR-I	ATP		P_2_XR	MNC	↑mEPSCs (amplitude)
Araque et al., 2002	R	ST	CA1	ACh	mAChR					
Bélair et al., 2010	F	VV	NMJ	ACh, ATP	mAChR, P_2_YR, P_2_XR					
Navarrete et al., 2012a,b	R	ST, VV	CA1	ACh	mAChR	Glu	mGluR		PY	↑synaptic release probability; LTP
Chen et al., 2012	M	VV, ST	V1	ACh	mAChR	n.r.		NMDAR	V1N	SICs, ↑neuronal firing
Perea and Araque, 2005	R	ST	CA1	ACh, Glu	mAChR, mGluR	Glu		NMDAR	PY	SICs
Bekar et al., 2008	M	VV	COR	NE	αAR					
Kulik et al., 1999	M	ST	CER^b^	NE	α_1_AR					
Gordon et al., 2005	R	VT, ST	PVN	NE	α_1_AR	ATP		P_2_X_7_R	MNC	↑mEPSCs (amplitude)
Min and Nevian, 2012	R	ST	BC	ECB^r^	CB_1_R	Glu	NMDAR		PY	↓EPSP (slope), t-LTD
Navarrete and Araque, 2010	M	ST	CA1	ECB^r^	CB_1_R	Glu	mGluR		PY	↑synaptic release probability
Navarrete et al., 2012a,b	H	ST	HIP, COR	n.r.	GluR, PR, CBR	Glu		NMDAR	PY	SICs
Navarrete and Araque, 2008	M	ST	CA1	ECB^r^	CB_1_R	Glu		NMDAR	PY	SICs
Panatier et al., 2006	R	ST	SON			D-ser		NMDAR	MNC	Control of long-term plasticity (metaplasticity)
Takata et al., 2011	M	VV	BC	ACh	mAChR	D-ser		NMDAR	PY	Control of LTP induction
Henneberger et al., 2010	R	ST	CA1	n.r.	n.r.	D-ser		NMDAR	PY	Control of LTP induction
Fellin et al., 2009	M	VV, ST	COR, HIP	n.r.	n.r.	D-ser		NMDAR	CN, PY	↑NMDAR-mediated currents
Yang et al., 2003	R	ST, VT	CA1	Glu	n.r.	D-ser		NMDAR	PY	Control of LTP induction
Lee et al., 2011	H	VT	COR			GABA				
Le Meur et al., 2012	R	ST	HIP			GABA, Glu		GABA_A_R, NMDAR	PY	SOCs (GABA_A_R); SICs (NMDAR)
Lee et al., 2010	M	ST	CER^b^			GABA		GABA_A_R^s^	GC, PF^s^	↑neuronal inhibition^s^
Kozlov et al., 2006	R	ST	OB			GABA, Glu		GABA_A_R, NMDAR	MC	SOCs (GABA_A_R); SICs (NMDAR)
Serrano et al., 2006	R	ST	CA1	GABA	GABA_B_R	ATP	A_1_R		PY	↓fEPSP (amplitude); heterosynaptic depression
Kang et al., 1998	R	ST	CA1	GABA	GABA_B_R	n.r.	iGluR	iGluR	PY	↑mIPSCs (frequency and amplitude)
Stellwagen and Malenka, 2006	M	VT	HIP			TNFα		n.r.	PY	↑AMPAR; LTP
Beattie et al., 2002	R	ST, VT	HIP			TNFα		n.r.	PY	↑AMPAR
Santello et al., 2011	M	ST, VT	MLDG			TNFα, Glu				Control of Glu exocytosis from astrocytes
Bezzi et al., 2001	R, H	ST, VT	HIP			TNFα, Glu				Control of Glu exocytosis from astrocytes
	

*Reference to in vitro studies is included whenever the latter are missing*.

1 *Specimen. F, frog; H, human; M, mouse; R, rat*.

2 *Preparation. VT, in vitro/cultures; ST, in situ/slices; VV, in vivo*.

3 *Brain area or body area. BC, barrel cortex; CA1, Cornu Ammonis area 1; CER, cerebellum; COR, cortex, DH, dorsal horn; HIP, hippocampus; MLDG, molecular layer dentate gyrus; NAcc, nucleus accumbens; NMJ, neuromuscular junction; PVN, paraventricular nucleus of the hypothalamus; Ret, retina; VBT, ventrobasal thalamus; V1, primary visual cortex; VC, visual cortex*.

4 *Synaptically-released neurotransmitter. Ach. acetylcholine; ECB, endocannabinoids; NE, norepinephrine*.

5 *Astrocytic receptors targeted by synaptically-released neurotransmitters (pathway A) and neuronal receptors targeted by gliotransmitters (pathways B and C). αAR (α_1_AR), α- (α_1_-) adrenergic receptors; CBR, cannabinoid receptors; iGluR, ionotropic glutamate receptors; KAR, kainate receptor; mAChR, muscarinic receptors; mGluR-I, mGluR-II/III, group I (II/III) metabotropic glutamate receptors*.

6 *Gliotransmitter released from astrocyte*.

7 Cell targeted by gliotransmitters, where targeted receptors and effects were reported. AC, amacrine cell; CN, cortical neuron; GC, granule cell; IN, interneuron; MF, muscle fiber; PF, parallel fiber axon; PY, pyramidal neuron; MNC, magnocellular neuroscretory cell; MSN, medium spiny neurons; SGN, substantia gelatinosa neuron; RN, retinal neuron; V1N, V1 excitatory neuron

8 *Effects triggered by gliotransmission on pathways B or C in Figure 1B. (f)EPSP, (field) excitatory postsynaptic potential; mEPSC (mIPSC), miniature excitatory (inhibitory) postsynaptic current; t-LTP (t-LTD), spike-timing-dependent long-term potentiation (depression); LTP, long-term potentiation; PTD, post-tetanic depression; PTP, post-tetanic potentiation; SICs, slow inward (depolarizing) currents; SOCs, slow outward (hyperpolarizing) currents. Arrows denote increase (↑) or decrease (↓) and are followed by the associated signal, e.g. ↑(↓)EPSP (frequency)(amplitude)(slope): increase (decrease) in (frequency)(amplitude) (slope) of EPSPs. fEPSP, field EPSP*.

Other: b, study on Bergmann glia cells; r, retrograde signaling; s, suggested by investigators; m, study on Müller glia cells; n.r., when existence of a specific signaling pathway is observed but details are not reported;

* *indirect evidence*.

Analysis of the scheme in Figure 1B reveals that astrocytes mediate two loops in the signal flow from presynaptic to postsynaptic terminal: a *feedforward* and a *feedback* one. The feedforward loop ends on the postsynaptic terminal and is activated when synaptic glutamate and/or ATP induces glutamate and/or D-serine release from the astrocyte to the postsynaptic element, i.e. the **A**–**C** path in Figure 1B (Bains and Oliet, 2007; Barres, 2008; Santello and Volterra, 2009). The feedback loop ends on the presynaptic terminal (the **A**–**B** path in Figure 1B) and is activated when synaptic glutamate or ATP trigger Ca^2+^-dependent release of glutamate and/or ATP from the astrocyte to the presynaptic terminal, leading to modulation of synaptic release through specific presynaptic receptors (Santello and Volterra, 2009; Halassa and Haydon, 2010).

In principle the two pathways could coexist at the same synapse where they are expected to display different dynamics and respond to different preferred input stimuli. Therefore their coexistence at the same synapse could give rise to complex effects that are hard to quantify when considered altogether. Accordingly, a common approach in experiments is to characterize their effects on synaptic function by separate manipulations of pathways **A–C** (Figure 1B) using different techniques (Montana et al., 2004; Jourdain et al., 2007; Marchaland et al., 2008; Di Castro et al., 2011; Panatier et al., 2011; Santello et al., 2011). For example, characterization of the feedback pathway on the presynaptic terminal (**A**–**B**) can be carried out by opening the feedback loop (for example by inhibiting elements of **A** or **B**) and analyzing the signaling components separately. From a theoretical point of view, this approach is put forth by at least three steps of analysis that are: (1) characterization of Ca^2+^ dynamics in the astrocyte as a function of different synaptic inputs (that is, pathway **A**); (2) characterization of how gliotransmitter release from the astrocyte depends on different astrocytic intracellular Ca^2+^ dynamics; and (3) characterization of the effect on synaptic release of Ca^2+^-dependent gliotransmitter release from the astrocyte (i.e. pathway **B**). These three aspects are discussed below from a modeling perspective, focusing on their possible roles in synaptic information processing.

## Characteristics of astrocyte Ca^2+^ excitability and its relationship with synaptic activity

Intracellular Ca^2+^ elevations in the astrocyte are not simple on–off signals (Carmignoto, 2000; Zonta and Carmignoto, 2002; Di Castro et al., 2011). There are multiple and varied spatiotemporal patterns of Ca^2+^ elevation, which probably underlie different types of function, including generation of diverse output signals (Carmignoto, 2000; Volterra and Meldolesi, 2005; Zorec et al., 2012). Two main types of neuronal activity-dependent Ca^2+^ responses are observed in astrocytes (Grosche et al., 1999; Codazzi et al., 2001; Matyash et al., 2001; Zonta and Carmignoto, 2002; Scemes and Giaume, 2006): (1) transient Ca^2+^ increases that are confined to their distal processes (Pasti et al., 1997; Nett et al., 2002; Di Castro et al., 2011) and (2) Ca^2+^ elevations propagating along these processes as regenerative Ca^2+^ waves, often eventually reaching the cell soma (Pasti et al., 1997; Sul et al., 2004). This latter kind of response can even propagate to neighboring astrocytes, giving rise to intercellular Ca^2+^ waves (Tian et al., 2005; Kuga et al., 2011). On the other hand, intercellular Ca^2+^ propagation does not necessarily need propagation through the cell soma and has been observed across astrocytic processes or from an end foot to an end foot (Mulligan and MacVicar, 2004; Giaume et al., 2010).

**Figure 2:**
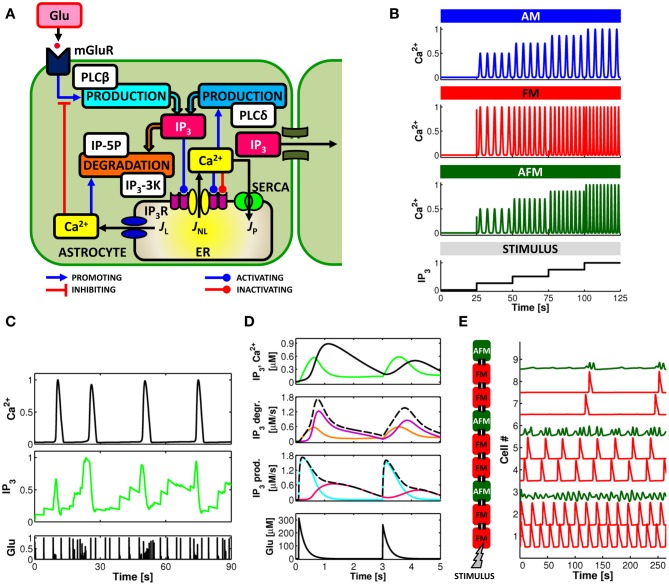
**Computational aspects of astrocytic Ca^2+^ signaling. (A)** Scheme of IP_3_-mediated Ca^2+^-induced Ca^2+^ release in the astrocyte. Calcium and IP_3_ signals are controlled by synaptic glutamate through metabotropic glutamate receptor- (mGluR-) PLCβ-mediated IP_3_ production (see text for details). **(B)** IP_3_ and Ca^2+^ signals can be envisioned to encode incoming synaptic activity through frequency and amplitude of their oscillations. Astrocytes could thus encode synaptic information either by modulations of the amplitude (AM), the frequency (FM), or both (AFM) of their Ca^2+^ oscillations. **(C)** Simulated Ca^2+^ and IP_3_ patterns in response to sample synaptic glutamate release (Glu) in a model astrocyte (De Pittà et al., 2009a,b) reveals that IP_3_ signals could be locked in the AFM-encoding independently of the encoding mode of the associated Ca^2+^ signals. This feature could allow the astrocyte to optimally integrate synaptic stimuli. (**D**, *top*) Simulated Ca^2+^ (*black trace*) and IP_3_ (*green trace*) signals in the same astrocyte model as in **(C)**, and associated rates of IP_3_ production (prod.) and degradation (degr.) (*middle panels*, *dashed black lines*) in response to two consecutive synaptic glutamate release events (*bottom panel*, Glu). The analysis of the contributions of different enzymes to IP_3_ signaling (*solid colored traces*; *cyan*: PLCβ ; *pink*: PLCδ ; *orange*: IP-5P; and *purple*: IP_3_-3K) reveals dynamical regulation by Ca^2+^ of different mechanisms of IP_3_ production/degradation which could ultimately underlie dynamical regulation of astrocyte processing of synaptic stimuli. **(E)** Simulated propagation of Ca^2+^ waves in a heterogeneous linear chain composed of both FM (*red traces*) and AFM (*green traces*) astrocytes reveals that encoding of synaptic activity (STIMULUS) could change according to cell location along the chain. Adapted from Goldberg et al. (2010).

The precise signaling cascades underlying the various forms of Ca^2+^ elevation are not completely understood. In general, Ca^2+^ signals in astrocytes are determined by an intricate interplay of amplification, buffering, and extrusion pathways linked to cytosolic Ca^2+^ elevations mediated by influx from the extracellular space (Malarkey et al., 2008; Shigetomi et al., 2011) and/or release from intracellular endoplasmic reticulum (ER) stores (Verkhratsky et al., 2012). IP_3_-triggered Ca^2+^-induced Ca^2+^ release (CICR) from the ER is considered the primary mechanism responsible for intracellular Ca^2+^ dynamics in astrocytes (Volterra and Meldolesi, 2005; Nimmerjahn, 2009). This mechanism, schematized in Figure 2A, is essentially controlled by the interplay of three fluxes: (1) a Ca^2+^ transfer from the cytosol to the ER (*J*_P_) mediated by endoplasmic-reticulum Ca^2+^-ATPase (SERCA) pumps which contributes to the maintenance of higher Ca^2+^ concentrations in the ER stores than in the cytosol; (2) a passive Ca^2+^ leak (*J*_L_) from the ER to the cytosol that is driven by the Ca^2+^ gradient between the ER and the cytosol; and (3) an efflux (*J*_NL_) from the ER to the cytosol through IP_3_ receptor (IP_3_R) channels, which depends both on IP_3_ and Ca^2+^ concentrations in the cytosol in a nonlinear fashion (Bezprozvanny et al., 1991; Ramos-Franco et al., 2000; Shinohara et al., 2011).

Cytosolic Ca^2+^ regulates IP_3_Rs in a biphasic manner: Ca^2+^ release from the ER is potentiated at low cytosolic Ca^2+^ concentrations but is inhibited at higher Ca^2+^ concentrations (Iino, 1990; Bezprozvanny et al., 1991). On the other hand, IP_3_ monotonically activates IP_3_R channels at constant Ca^2+^ concentrations (Watras et al., 1991), but dynamically changes the Ca^2+^ sensitivity of the channel (Kaftan et al., 1997; Ramos-Franco et al., 2000; Mak et al., 2003). At low, subsaturating IP_3_ concentrations, the optimal Ca^2+^ concentration for IP_3_R modulation becomes lower, whereas at very high IP_3_ concentrations, channel activity persists at supramicromolar Ca^2+^ concentrations (Kaftan et al., 1997; Mak et al., 2003). Thus, the level of IP_3_ determines the dynamics of intracellular Ca^2+^.

Both production and degradation of IP_3_ depend on enzymes that are regulated by cytosolic Ca^2+^ (Berridge et al., 2003; De Pittà et al., 2009a,b). These include Ca^2+^-dependent PLCδ-mediated IP_3_ synthesis and Ca^2+^-dependent IP_3_ degradation by IP_3_ 3-kinase (IP_3_-3K) and by inositol polyphosphate 5-phosphatase (IP-5P) (Figure 2A) (Zhang et al., 1993; Sims and Allbritton, 1998; Rebecchi and Pentyala, 2000). However, while the activity of IP_3_-3K is stimulated by cytosolic Ca^2+^ (Communi et al., 1997), IP-5P is inhibited instead (Communi et al., 2001). This results in different mechanisms of IP_3_ degradation depending on the Ca^2+^ concentration in the cytoplasm (Sims and Allbritton, 1998; Irvine et al., 2006). Thus, for example, for equally-expressed enzymes, at low Ca^2+^ concentrations, namely lower than 500 nM (Sims and Allbritton, 1998; De Pittà et al., 2009a,b), IP_3_ degradation is promoted by both IP-5P and IP_3_-3K, whereas for intermediate-to-high cytosolic Ca^2+^ concentrations, degradation by IP_3_-3K becomes predominant (Sims and Allbritton, 1998). Theoretical investigation showed that the interplay of these two regimes is both necessary and sufficient to reproduce Ca^2+^ oscillations and pulsations observed experimentally (De Pittà et al., 2009a,b).

Intracellular levels of IP_3_ can also be controlled by gap junction mediated diffusion of IP_3_ from other regions of the same astrocyte or from neighboring cells (Giaume et al., 2010) (i.e., the I/O pathway in Figure 1B). Moreover, synaptic glutamate (or other synaptic agents) can bind to astrocytic G protein-coupled receptors (GPCRs) like mGluRs that are directly linked to intracellular IP_3_ production by PLCβ (Zur Nieden and Deitmer, 2006) (pathway **A** in Figure 1B). In this fashion, astrocytic Ca^2+^ dynamics triggered by synaptically-controlled IP_3_ production can be regarded as a form of encoding information about activity in neighboring synapses.

Encoding of synaptic activity by astrocytic Ca^2+^ is likely multimodal, depending on many possible intracellular properties (De Pittà et al., 2008, 2009a,b; Dupont et al., 2011). A widely adopted classification considers the amplitude and the frequency of Ca^2+^ increases from resting levels (Berridge, 1997; Falcke, 2004; De Pittà et al., 2008, 2009a,b). In this view, as summarized in Figure 2B, synaptic activity reflected by different intracellular IP_3_ concentrations (STIMULUS), is encoded by the modulation of Ca^2+^ oscillations and pulsations either in their amplitude (AM), their frequency (FM), or both (AFM). While available experimental data suggest a preferential FM mode of encoding (Pasti et al., 1997), AM and AFM encoding of synaptic activity are also plausible mechanisms given that the amplitude of Ca^2+^ response can strongly depend on the stimulation intensity (Wang et al., 2006; Di Castro et al., 2011; Panatier et al., 2011; Torres et al., 2012). This is the case for example, of synaptic inputs that occur rapidly one after the other whereby the ensuing intracellular Ca^2+^ concentration builds up as a cumulation of such inputs (Perea and Araque, 2005; Torres et al., 2012). In further support of the AM/AFM encoding is the experimental observation that glutamate exocytosis from the astrocyte occurs only when Ca^2+^ increases beyond a threshold concentration (Newman and Zahs, 1997; Parpura and Haydon, 2000; Pasti et al., 2001; Auld and Robitaille, 2003; Montana et al., 2006). Hence, astrocytic Ca^2+^ increases in response to synaptic activity would not systematically trigger the release of glutamate or other gliotransmitters from the astrocyte. Acting on the amplitude of astrocytic Ca^2+^ signals, AM/AFM encodings could constitute a way to regulate astrocytic gliotransmitter release by synaptic activity. Further experiments are needed to elucidate the nature of the Ca^2+^ threshold for astrocytic exocytosis since this latter might be gliotransmitter-specific (Montana et al., 2006). Accordingly, AM/AFM encoding of Ca^2+^ dynamics could vary from one gliotransmitter to another.

Experimental evidence suggests that Ca^2+^ dynamics does not simply mirror synaptic activity but is more complex, to a point that astrocytes have been proposed to perform genuine processing of synaptic information (Perea and Araque, 2005; Perea et al., 2009). This possibility follows from the complex network of IP_3_ and Ca^2+^ signaling and subtends a scenario where Ca^2+^ could be only one of the players in the encoding and processing of synaptic activity by astrocytes (Mishra and Bhalla, 2002). Yet many, if not all, of the other signals underlying the complex cascade of biochemical reactions that link synaptically-released glutamate to CICR, could also carry out encoding and processing (Barlow, 1996; Berridge et al., 2003). Theoretical investigations suggested that IP_3_ could also encode for the glutamate stimulation levels via a systematic AFM encoding (De Pittà et al., 2009a,b) as shown in Figure 2C. When cytosolic Ca^2+^ levels are low, close to resting values, IP_3_ generally increases with ongoing synaptic activity (Glu). With low Ca^2+^, the activity of IP_3_-3K is reduced and the resulting IP_3_ degradation slows down. The contribution to IP_3_ production by Ca^2+^-dependent PLCδ is reduced as well, so that intracellular IP_3_ mostly depends on the frequency of synaptic release. Rapid successions of synaptic release events produce crisp increases of IP_3_ (essentially proportional to the number of successive synaptic release events) while, between two remote release events, IP_3_ tends to relax to resting levels. As a result, IP_3_ dynamics overall evolves as the integral of synaptic activity. If IP_3_ reaches the CICR-triggering threshold, intracellular Ca^2+^ increases fast and so does IP_3_-3K activity. Then, IP_3_ is rapidly degraded and resting IP_3_ levels are restored, thus resetting the integral of synaptic activity to initial values.

It is precisely the alternation between these two different phases of IP_3_ degradation (a high Ca^2+^—high IP_3_-3K-activity phase and a low Ca^2+^—low IP_3_-3K-activity phase), that endows IP_3_ signal with high amplitude variability. On the other hand, such AM features still allow fast variations, thus rich spectral content (i.e., FM features), in response to changes in frequency of synaptic release. This enticing possibility could endow the IP_3_ signal with the necessary properties to function as optimal interface between synaptic stimuli and intracellular Ca^2+^ signals. Since neural information is carried by the timing of spikes rather than by their amplitude (Sejnowski and Paulsen, 2006), the capability of fast highly-variable amplitude changes corresponding to rich spectral content of IP_3_ signals, would fulfill this requirement, embedding the essential spectral features of the synaptic signal into the spectrum of the IP_3_ transduction. On the other hand, because Ca^2+^ signals are triggered primarily by suprathreshold IP_3_ elevations (Li et al., 1994; Keizer et al., 1995), the coexistence of AM features within the AFM IP_3_ signal seems to be a necessary prerequisite in order to trigger CICR.

This could also help elucidate the origin of the integrative properties of Ca^2+^ signaling in astrocytes (Perea et al., 2009). These properties could result from at least two steps of integration: one step is the transduction of the agonist signal into the IP_3_ signal; the other step is the cross-talk between IP_3_ and Ca^2+^ signals. Hence, AFM-encoding IP_3_ dynamics could operate a first preliminary integration by smoothing the highly indented synaptic stimulus. The inherent features of CICR would then bring forth a further integration step, yielding Ca^2+^ patterns that are even smoother than IP_3_ signals (De Pittà et al., 2009a,b).

The tight dynamical coupling between IP_3_ and Ca^2+^ signals also suggests that the way astrocytes process synaptic signals, i.e. their frequency response to synaptic stimuli, is not fixed but rather dynamical and dependent on the history of activation of the astrocyte. This is because different IP_3_ signaling mechanisms that are dynamically regulated by Ca^2+^ likely correspond to different frequency responses of the astrocyte with respect to synaptic signals. Figure 2D shows the time course of IP_3_ production (IP_3_ prod.) and degradation (IP_3_ degr.) (*dashed black lines*) underlying simulated IP_3_ and Ca^2+^ signals (*top panel*, *green* and *black traces*, respectively) in response to two events of synaptic glutamate release (*bottom panel*). While Ca^2+^-dependent IP_3_ production by PLCβ (*cyan trace*) and PLCδ (*pink trace*) could modulate the threshold frequency of synaptic stimuli that triggers Ca^2+^ signaling in the astrocyte, existence of different regimes of IP_3_ degradation within a single Ca^2+^ oscillation cycle could be responsible for different cutoff frequencies of synaptic release beyond which Ca^2+^-mediated astrocyte processing of synaptic stimuli ceases. In particular, the cutoff frequency during low IP_3_-3K activity could be mainly set by the rate of IP_3_ degradation by IP-5P (*orange trace*). When Ca^2+^ is high instead, IP_3_ degradation by IP_3_-3K (*purple trace*) could also become very strong, thus sensibly reducing the cutoff frequency. That is, the cutoff frequency is dependent on the context of underlying Ca^2+^ signaling which, in turn, depends on the history of activation of the astrocyte by synaptic stimuli.

The subcellular arrangement of the enzymes underlying IP_3_ signaling could also be responsible for spatial heterogeneity of the frequency response of the astrocyte. Although the subcellular localization of IP_3_ production and degradation enzymes in astrocytes remains to be elucidated, studies in brain tissue suggest that PLCβ and IP-5P could localize mainly in proximity to the plasma membrane, whereas PLCδ and IP_3_-3K are preferentially in the cytoplasm (Rebecchi and Pentyala, 2000; Irvine et al., 2006). Given that the ER distribution changes from astrocytic processes to soma (Pivneva et al., 2008), different subcellular regions of the astrocyte could correspond to different cytoplasmic volumes and thus to distinctly different expressions of enzymes mediating IP_3_ signaling. The ensuing different subcellular arrangement of these enzymes could ultimately provide anatomical specificity to the astrocytic phosphoinositide signaling which underlies CICR-based astrocyte processing of synaptic activity (Fukaya et al., 2008). In this fashion, processing of synaptic stimuli by Ca^2+^ signaling at astrocytic processes could differ from that carried out in the soma by means of differently expressed IP_3_ signaling-related enzymes.

## Computational aspects of propagating Ca^2+^ signals

Intracellular and intercellular propagation of Ca^2+^ could contribute new encoding and processing modes, in addition to those depicted in Figure 2B. However, despite the numerous modeling studies developed to account for the rich dynamics of astrocyte Ca^2+^ signaling (Bennett et al., 2008; Goldberg et al., 2010; Dupont et al., 2011); [for a recent review on calcium modeling see Falcke (2004)], we still lack a comprehensive theoretical framework to link the local Ca^2+^ signals that are restricted to small regions of the astrocytic processes, to their spatial dynamics and their possible propagation at larger spatial scales: intracellular propagation, global whole-cell signals or cell-to-cell Ca^2+^ waves.

Propagations at these various scales probably differ by their underlying mechanisms (Falcke, 2004; Scemes and Giaume, 2006). Fast-rising and short-lived local Ca^2+^ events, observed in response to even a single quantal release from synaptic terminals (Di Castro et al., 2011; Panatier et al., 2011) closely resemble spatially confined Ca^2+^ puffs or blips in other cell types (Thomas et al., 2000; Bootman et al., 2001) and could depend on spatial clustering of IP_3_Rs along the ER structures or of mGluRs along the plasma membrane, or on both (Marchaland et al., 2008; Panatier et al., 2011; Arizono et al., 2012). The cumulative recruitment of these Ca^2+^ puffs could lead to spatially more extended Ca^2+^ events which could either be still confined within astrocytic processes (Di Castro et al., 2011) or propagate to other cellular regions or to other cells as regenerative Ca^2+^ waves (Pasti et al., 1997; Kuga et al., 2011).

Calcium could propagate by at least two routes [for a recent review see Scemes and Giaume (2006)]. One is intracellular, through GJCs, involving diffusion of IP_3_ directly from cytoplasm to cytoplasm. The other route is extracellular, involving release of ATP from the astrocyte which binds to GPCRs of the same cell or neighboring astrocytes, increasing their IP_3_ levels (Guthrie et al., 1999). The relative contribution of each of these pathways likely depends on developmental, regional and physiological states and could subtend different ranges of propagations as well as different temporal features (Haas et al., 2006; Scemes and Giaume, 2006; Giaume et al., 2010).

The restriction and clustering of mGluRs expression along astrocytic processes to subregions that colocalize with synaptic terminals (van den Pol et al., 1995; Arizono et al., 2012) hints the possibility of a subcellular compartmentalization of Ca^2+^ signals (Marchaland et al., 2008; Di Castro et al., 2011; Panatier et al., 2011). Local Ca^2+^ events would be spatially restricted to narrow regions around each mGluRs cluster thus defining independent signals within the same process. In this fashion, astrocytes could carry out parallel integration and processing of synaptic information on different temporal and spatial scales at different processes (Goldberg et al., 2010; Bernardinelli et al., 2011) or even in different subregions of the same process which could constitute separate functional microdomains (Panatier et al., 2011). Furthermore, the resulting neuromodulatory action exerted by astrocytic gliotransmitters on synaptic terminals and their impact on neuronal network activity could vary from one process or microdomain to the other (Navarrete and Araque, 2011).

The spatial scale and the time window of Ca^2+^ events likely discriminate between different mechanisms of IP_3_ and Ca^2+^ signaling. At the level of astrocytic processes and subcellular compartments, Ca^2+^ propagation could be mediated by fast intracellular IP_3_ linear (i.e. Fickian) diffusion (Sneyd et al., 1994; Falcke, 2004). On the other hand, when considering intercellular propagation mediated by gap junctions, IP_3_ transport from one cell to the other could be essentially nonlinear. This scenario was shown to be consistent with the observed variability of Ca^2+^ wave propagation distance (Goldberg et al., 2010) and could explain the long-range regenerative propagation of Ca^2+^ waves observed in cortical astrocytes (Scemes and Giaume, 2006; Tian et al., 2006).

Cell heterogeneity likely constitutes an additional critical aspect that substantially affects propagation patterns and extent of propagation of Ca^2+^ signals (Iacobas et al., 2006; De Pittà et al., 2008; Goldberg et al., 2010). This possibility is illustrated in Figure 2E by a toy example consisting of a heterogeneous linear chain of FM-encoding (*red traces*) and AFM-encoding (*green traces*) astrocytes. The synaptic stimulus is restricted to the first cell of the chain (i.e. cell number 1). Only FM-encoding cells guarantee regenerative propagation of Ca^2+^ signals, whereas AFM cells do not, acting like propagation barriers along the chain. Unlike in AFM cells, Ca^2+^-dependent IP_3_ production in FM cells guarantees IP_3_ diffusion to the next cell in the chain to levels that are beyond the threshold of CICR thus promoting regenerative propagation (Goldberg et al., 2010). Moreover, the shape of the local Ca^2+^ events in each cell changes along the propagation path: after each AFM cell, the frequency of FM-encoding Ca^2+^ pulses is reduced, suggesting that different propagation patterns could carry out different processing of synaptic information (Goldberg et al., 2010).

The above scenario hints that the spatial distribution of astrocytes in different brain areas could be made to fulfill specific processing tasks. Indeed neighboring astrocytes in the brain are believed to be distributed in space in a non-random orderly fashion called “contact spacing” (Chan-Ling and Stone, 1991; Volterra and Meldolesi, 2005) or “tiling,” where each astrocyte creates its micro-anatomical domain with its processes overlapping with adjacent astrocytes only at their periphery (Bushong et al., 2002). Such spatial arrangement, combined with the heterogeneity of astrocytic responses, could be important in intercellular Ca^2+^ wave propagations and the related computational tasks carried out by astrocyte networks. The latter could be relevant in particular for the emergence of astrocytic functional maps observed in several brain areas (Giaume et al., 2010).

Increasing evidence argues in fact for a functional organization of astrocytes, reminiscent of that of cortical neurons (Bernardinelli et al., 2011). In the ferret visual cortex astrocytes, like neurons, respond to visual stimuli, with distinct spatial receptive fields and sharp tuning to visual stimulus features, including orientation and spatial frequency (Schummers et al., 2008). The stimulus-feature preferences of astrocytes there can be mapped across the cortical surface, in close register with neuronal maps (Schummers et al., 2008). Similar observations were also reported for astrocytes in the motor cortex (Haas et al., 2006) and in the somatosensory cortex (Schipke et al., 2008) as well as in the olfactory bulb (De Saint Jan and Westbrook, 2005).

Anatomical compartmentalization of astrocyte networks could underlie such functional organization. Both in the somatosensory barrel cortex and in the olfactory bulb, astrocytes are preferentially connected by gap junctions within the same barrel or olfactory glomerulus rather than between adjacent barrels or glomeruli (Houades et al., 2008; Roux et al., 2011). However, spatial confinement of gap junctions within single barrels/glomeruli might not fully account for the selective activation of astrocyte by electrical stimulation. Indeed, similar Ca^2+^ signals in response to the same stimulus could be observed in the same barrel yet with pharmacological block of astrocyte gap junctions (Schipke et al., 2008). Thus, additional factors must contribute to the astrocyte tuned response, which likely emerges as a result of the dynamical interactions with surrounding neurons (Rouach et al., 2004) and could ultimately depend on location and nature of activated cells among the other astrocytes in the network (Matyash and Kettenmann, 2010; García-Marqués and Lópes-Mascaraque, 2012). In the case of Figure 2E, for example, stimulated FM-encoding cells could trigger Ca^2+^ signaling in neighboring cells in a regenerative fashion thus extending their tuned response in space. On the other hand, AFM cells, acting as propagation barriers, could shape the borders of this tuned response, eventually drawing the topographical features of the ensuing functional map (Lallouette and Berry, 2012).

## Relating Ca^2+^ signals to gliotransmitter exocytosis from the astrocyte

There is a number of possible routes by which astrocytes could release gliotransmitters (Ni et al., 2007; Parpura and Zorec, 2010; Zorec et al., 2012), but Ca^2+^-dependent exocytosis is likely the major one on a physiological basis (Barres, 2008; Parpura et al., 2011). However, the identity of incoming inputs, the underlying molecular mechanism and the physiological conditions that govern gliotransmitter exocytosis largely remain to be elucidated (Montana et al., 2006; Ni et al., 2007; Santello and Volterra, 2009; Parpura and Zorec, 2010). Calcium-dependent exocytosis of glutamate or ATP from astrocytes, for example, may strictly depend on the nature of the upstream Ca^2+^ signal (Perea and Araque, 2005; Li et al., 2008; Marchaland et al., 2008; Pryazhnikov and Khiroug, 2008; Malarkey and Parpura, 2011); including the type of neurotransmitter involved and the type of receptor engaged (Enkvist and McCarthy, 1992; Muyderman et al., 2001; Coco et al., 2003; Bezzi et al., 2004; Blomstrand and Giaume, 2006). The influence of astrocytic glutamate or ATP on synaptic activity also likely depends both on the type of incoming stimulus and on the specific localization of the engaged receptor in the astrocyte (Perea and Araque, 2005; Santello and Volterra, 2009).

**Figure 3:**
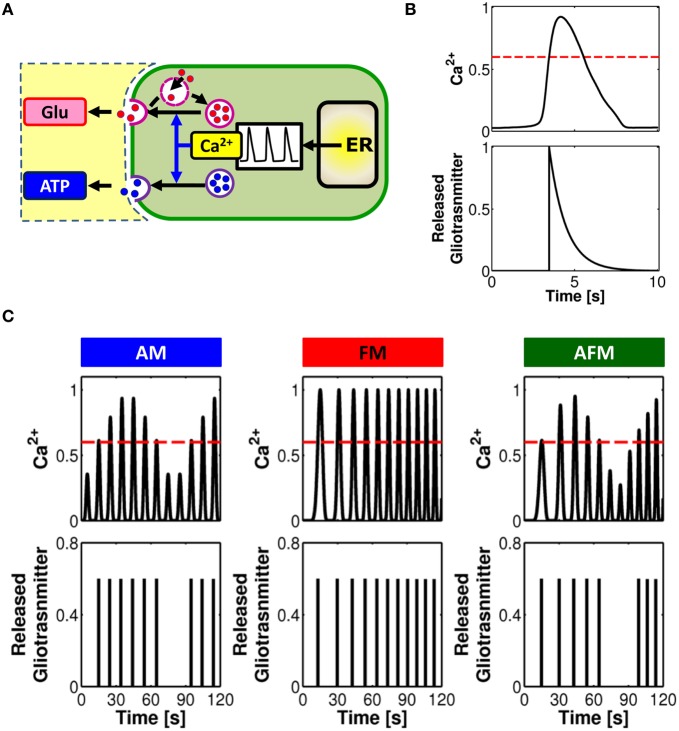
**Linking gliotransmitter exocytosis to various Ca^2+^ encoding modes. (A)** Calcium-dependent glutamate and ATP exocytosis from astrocytes are both brought forth by a vesicular compartment in the astrocyte competent for regulated exocytosis. The frequency of exocytotic events is directly controlled by the shape and frequency of Ca^2+^ oscillations. **(B)** Modeling concept for an “exocytosis event” from the astrocyte. Calcium (*top trace*) triggers exocytosis of glutamate or ATP every time it increases beyond a certain threshold concentration value (*red dashed line*). The overall release can then be approximated, under proper assumptions, by an exponentially-decaying pulse of extracellular concentration of glutamate or ATP (*bottom trace*). **(C)** Distinct Ca^2+^ encoding patterns could translate into distinct rates of gliotransmitter exocytosis events. In this way, synaptic activity encoded by astrocytic Ca^2+^ signals is linked to the frequency of glutamate/ATP release from the astrocyte in a unique fashion. Adapted from De Pittà et al. (2011).

A large amount of evidence suggests that gliotransmitter exocytosis from astrocytes bears several similarities with its synaptic homologous (Bergersen and Gundersen, 2009; Santello and Volterra, 2009; Bergersen et al., 2012) (Figure 3A). Astrocytes possess vesicular compartments that are competent for the regulated exocytosis of glutamate (Bezzi et al., 2004; Bergersen and Gundersen, 2009) and ATP (Coco et al., 2003; Jaiswal et al., 2007; Zhang et al., 2007). Similarly to synapses, astrocytes express soluble N-ethylmaleimide-sensitive factor attachment protein receptors (SNARE) necessary for exocytosis (Parpura et al., 1995; Schubert et al., 2011) as well as proteins responsible for concentrating glutamate or ATP into vesicles (Bezzi et al., 2004; Montana et al., 2004; Zhang et al., 2004b; Sawada et al., 2008). Fusion with the plasma membrane, trafficking and recycling of astrocytic glutamate and ATP secretory vesicles have been observed (Bezzi et al., 2004; Chen et al., 2005; Crippa et al., 2006; Jaiswal et al., 2007; Pangršic et al., 2007; Stenovec et al., 2007), which are indicative of quantal glutamate and ATP release (Del Castillo and Katz, 1954; Pasti et al., 2001; Domercq et al., 2006; Jaiswal et al., 2007; Pangršic et al., 2007; Marchaland et al., 2008; Santello et al., 2011).

Glutamate exocytosis from cultured astrocytes evoked by GPCRs is observed with short delay, i.e. 50–100 ms, after the rise in submembrane Ca^2+^, and is mediated by a rapid succession of fusion events which peaks within ~500 ms from the onset and decays to baseline much more slowly (>1 s), though generally before the recovery of basal Ca^2+^ levels (Domercq et al., 2006; Marchaland et al., 2008; Santello et al., 2011) (refer also to Table 2). In addition to this “exocytotic burst,” larger delays with slower rate of sustained vesicular fusion events have also been reported (Kreft et al., 2004; Malarkey and Parpura, 2011). The delayed onset with respect to the Ca^2+^ rise is consistent with a threshold Ca^2+^ concentration for release (Pasti et al., 1997; Parpura and Haydon, 2000). On the other hand the overall release of glutamate is characterized by a rising phase much faster than its decay and can be approximated by an exponential function like synaptic exocytosis yet with a decay time much slower than the latter (Marchaland et al., 2008; De Pittà et al., 2011; Santello et al., 2011) (Figure 3B). Although much less characterized than its glutamate counterpart, astrocytic ATP exocytosis could occur in a qualitatively similar fashion in spite of different underlying kinetics (Pangršic et al., 2007; Pryazhnikov and Khiroug, 2008; Li et al., 2008). Based on these arguments, it was proposed that astrocytic gliotransmitter release could be modeled using the same mathematical description of synaptic release, although the two mechanisms are likely different in their molecular machinery, with the kinetics of astrocyte release much slower than synaptic release (De Pittà et al., 2011; Schubert et al., 2011).

**Table 2:** **Comparison of time scales of rise, decay and full-width half-maximum (FWHM) duration of changes of intracellular Ca^2+^, extracellular glutamate (Glu) and ATP in astrocytes and neurons**.

**Signal**	**Origin**	**τ_rise_ [s]**	**τ_decay_ [s]**	**FWHM [s]**	**Essential references**
Ca^2+^	Astrocyte (soma)	2–20^f^	3–25^f^	5–160	Hirase et al., 2004; Nimmerjahn et al., 2004; Wang et al., 2006
	Astrocyte (soma)^a^	~0.5	~1.1	~2–4	Winship et al., 2007
	Neuron (soma)	2–5·10^−3^	0.1–0.4	~0.1–0.3	Svoboda et al., 1997; Waters et al., 2003; Nimmerjahn et al., 2004
	Astrocyte (process)	0.1–0.2	0.2–4^f^	~0.5–4	Di Castro et al., 2011; Panatier et al., 2011
	Neuron (presynaptic bouton)	0.5–5·10^−3^	~0.1–2	0.1–1^*^	Regehr et al., 1994; Emptage et al., 2001
Glu	Astrocyte	0.2–0.5	0.5–1.5	1–6	Domercq et al., 2006; Marchaland et al., 2008; Santello et al., 2011
	Neuron (synapse)	1–5·10^−4^	0.01–0.1	0.01–0.1^*^	Raghavachari and Lisman, 2004; Herman and Jahr, 2007; Okubo et al., 2010
ATP	Astrocyte	0.1–0.5	2–3	2–20	Pangršic et al., 2007; Li et al., 2008
	Neuron (synapse)	1–5·10^−4^^*^	0.2–1^*^	0.15–0.5	Dundwiddie and Masino, 2001; Pankratov et al., 2007

Glutamate and ATP values refer to transient increases of their extracellular concentrations following release in a Ca^2+^-dependent fashion. Therefore they describe the time course of the overall glutamate and ATP released by an exocytotic burst rather than by a single exocytotic event which can be much faster and occur within the first ~50 ms from Ca^2+^ rise (see text). Indicative rise and decay time constants as well as FWHM values are reported in terms of min–max ranges. Fast calcium signals imaging in astrocyte in the somatosensory cortex reported by Winship et al. (2007) are reported separately and dubbed by “a”. The letter “f” stands for values that were obtained by fitting of experimental data by a biexponential function such as f(t) = C·(exp(–t/τ_decay_) – exp(–t/τ_rise_)) with C being a proper scaling factor. Asterisk “

* *” denotes values estimated by a model of astrocytic and synaptic release introduced in De Pittà et al. (2011)*.

How are different modes of Ca^2+^ encoding translated into glutamate or ATP release? Assuming proper conditions about the rate of clearance of these gliotransmitters with respect to the underlying intracellular Ca^2+^ dynamics that mediate their exocytosis (Abbracchio et al., 2009), an intriguing theoretical prediction is that various patterns of Ca^2+^ oscillations could mostly correspond to different rates of gliotransmitter release (De Pittà et al., 2011). This is presented in Figure 3C where, for three stereotypical patterns of Ca^2+^ oscillations, that is AM, FM, and AFM, the corresponding timing of gliotransmitter release from the astrocyte is shown. In this example, FM-encoding Ca^2+^ oscillations always cross the threshold for exocytosis (*dashed red line*), triggering gliotransmitter release every time. Conversely, AM or AFM oscillations may not be large enough to reach such threshold, resulting in some oscillations that fail to trigger gliotransmitter release. In this fashion, while FM Ca^2+^ oscillations trigger gliotransmitter exocytosis at their own frequency, the amplitude of AM and AFM oscillations could selectively discriminate which Ca^2+^ pulse triggers exocytosis, eventually dictating the frequency of “measured” glutamate or ATP release events (De Pittà et al., 2011). Further experimental investigations are required to elucidate whether such prediction could effectively mimic reality.

While astrocytic Ca^2+^ signals could be triggered both by spontaneous and evoked synaptic release, gliotransmitter release might be not (Di Castro et al., 2011; Panatier et al., 2011). Blockade of Ca^2+^-dependent glutamate release in astrocytes in the dentate gyrus was indeed observed to be effective in reducing the frequency of synaptic release events only when these were evoked by action potentials, but not when they happened spontaneously. This suggests that release of glutamate from astrocytes in this region could effectively occur only in presence of evoked synaptic activity (Di Castro et al., 2011). Interestingly, the Ca^2+^ elevations in astrocyte processes triggered by action potentials were reported to be more complex, larger in amplitude and more extended in space, than those generated by spontaneous synaptic release events. Since glutamate could be released from several sites along the same astrocyte process and this latter could contact several synapses (Panatier et al., 2011), one of such Ca^2+^ elevations could then generate multiple spatially-distinct glutamate release events modulating synaptic release at several other synapses. The same Ca^2+^ elevation however, could bring forth at subcellular regions of the process, different local Ca^2+^ dynamics, in close analogy to the behavior of the heterogeneous AM/AFM oscillations in the astrocyte chain in Figure 2E. Such different local Ca^2+^ dynamics could in turn result in different rates of glutamate release and thus in different modulations of synaptic release in a non-random fashion. In this way, the same astrocytic process or a segment of it, could carry out multiple regional modulations of synaptic release, depending both on the temporal and spatial dynamics of synaptic release. It is however possible that the mechanisms underlying Ca^2+^-dependent gliotransmission differ in different brain areas (Matyash and Kettenmann, 2010; Zhang and Barres, 2010). Indeed, in contrast with the above observations in the dentate gyrus, astrocytes in the stratum radiatum of the hippocampus were reported to release glutamate even in the absence of synaptic activity evoked by action potentials (Bonansco et al., 2011).

Another issue is what makes a single astrocyte release one gliotransmitter rather than another. A possibility is that different gliotransmitters are released in response to different stimuli. *In situ* studies indeed suggest that purinergic GPCR-mediated astrocytic Ca^2+^ signals could preferentially trigger glutamate release (Jourdain et al., 2007; Perea and Araque, 2007; Di Castro et al., 2011) (see also Table 1). In contrast, Ca^2+^ elevations triggered by glutamate could bring forth ATP release (Pascual et al., 2005; Gordon et al., 2009; Panatier et al., 2011). Overall these observations hint that a tight association likely exists between the type of targeted astrocytic receptor and the secretory machinery of gliotransmitters (Zorec et al., 2012). Moreover, in the same astrocyte, different gliotransmitters could be contained in different organelles with different secretory properties in response to Ca^2+^ signals (Coco et al., 2003). Indeed, while glutamate seems to be preferentially released by synaptic-like microvesicles (Bezzi et al., 2004; Jourdain et al., 2007; Bergersen et al., 2012), ATP is likely released by dense-core granules (Coco et al., 2003), and/or lysosomes (Jaiswal et al., 2007; Zhang et al., 2007; Li et al., 2008). Although the underlying molecular machinery of exocytosis remains to be elucidated, each organelle population is likely secreted in a different fashion (Pryazhnikov and Khiroug, 2008). *In vitro* evidence showed in fact that Ca^2+^ signals that triggered release of glutamate-containing vesicles (Marchaland et al., 2008) did not release ATP-filled organelles and vice versa (Coco et al., 2003; Li et al., 2008), ultimately suggesting that glutamate and ATP could be released in response to different Ca^2+^ signals (Parpura and Zorec, 2010).

Intriguingly, stimulation of astrocyte GPCRs can evoke fast gliotransmitter exocytosis within few hundreds of milliseconds (Bezzi et al., 2004; Domercq et al., 2006; Marchaland et al., 2008; Santello et al., 2011), indicating that the Ca^2+^-dependent process that couples stimulus with secretion must be fast. Indeed, the peak of GPCR-mediated Ca^2+^ release from the ER can be as fast as 50–250 ms (Marchaland et al., 2008; Di Castro et al., 2011; Panatier et al., 2011) and Ca^2+^-dependent exocytosis of single glutamate vesicles can occur within less than 5–20 ms from Ca^2+^ elevation (Chen et al., 2005; Bowser and Khakh, 2007; Marchaland et al., 2008; Santello et al., 2011). Therefore, to assure fast stimulus-secretion coupling, IP_3_ diffusion from the site of production by GPCRs at the plasma membrane to IP_3_ receptors on the ER membrane must also be of the order of tens of milliseconds and so must Ca^2+^ diffusion from the mouth of IP_3_Rs to the Ca^2+^ sensor of exocytosis (Zhang et al., 2003, 2004a; Ni et al., 2007). Given that the diffusion times of IP_3_ or Ca^2+^ can be estimated as the half of the square of the diffusion distance from the site of their production in the cytoplasm divided by the respective diffusion constants (Syková and Nicholson, 2008), which can be as high as ~200–300 μm^2^/s for IP_3_ (Allbritton et al., 1992; Sneyd et al., 1994) and 30 μm^2^/s for Ca^2+^ (Kang and Othmer, 2009), then diffusion times of the order of milliseconds could be obtained only for diffusion lengths at most in the micron range. This prediction is fully supported by the functional evidence of local GPCR-evoked, ER-dependent Ca^2+^ microdomains in astrocytic processes (Di Castro et al., 2011; Panatier et al., 2011) and by the morphological evidence that at astrocytic processes the ER stores are found at ~200–600 nm distance both from the plasma membrane and gliotransmitter-containing vesicles (Bezzi et al., 2004; Marchaland et al., 2008; Bergersen et al., 2012). Moreover, GPCR agonist-mediated Ca^2+^ transients were observed restricted areas beneath the plasma membrane, in close proximity to the sites of exocytosis (Marchaland et al., 2008), suggesting that both GPCRs and secretory vesicles must be spatially close to the IP_3_Rs responsible for Ca^2+^ release from the ER. This indicates that the location of IP_3_Rs along the ER membrane in astrocyte processes might be non-random (Blaustein and Golovina, 2001), in tight spatial association with astrocytic GPCRs on the plasma membrane and releasable gliotransmitter organelles (Marchaland et al., 2008; Panatier et al., 2011). Such spatial coupling could ultimately underlie the preferential occurrence of functional gliotransmission from astrocytic processes rather than from the soma (Gordon et al., 2009), entailing strict spatiotemporal requirements for Ca^2+^ signals to trigger release of gliotransmitters from the astrocyte and modulate synaptic transmission.

## Modulation of synaptic release by astrocytic glutamate and ATP

**Figure 4:**
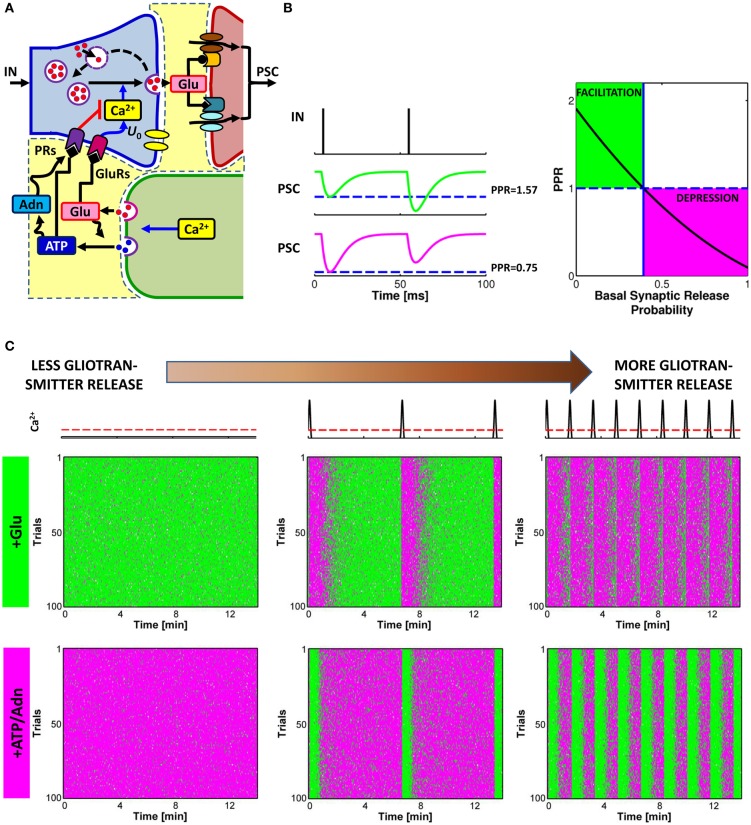
**Glutamate or ATP released from astrocytes regulates transitions between facilitation and depression of synaptic transmission. (A)** Conceptual framework for the regulation of synaptic release probability at basal conditions by astrocytes. Astrocyte-released glutamate increases basal synaptic release probability (*U*_0_), whereas astrocyte-released ATP/Adn generally decreases it. **(B)** Changes in synaptic release probability due to astrocytic gliotransmitters can be detected by variations of paired-pulse plasticity quantified by paired-pulse ratio (PPR). Paired-pulse facilitation (*left*, *green traces*) of postsynaptic currents (PSCs) corresponds to PPR values above 1 (*right*, *green-shaded area*) whereas paired-pulse depression (*left*, magenta traces) are associated with PPR values below 1 (*right*, *magenta-shaded area*). **(C)** Raster plots of simulated PSC pairs for 100 different input spike trains with same statistics colored according to the paired-pulse ratio: *green* for facilitation, PPR > 1; *magenta* for depression, PPR < 1. For increasing rates of exocytosis of gliotransmitter from the astrocyte, mimicked by increasing rates of Ca^2+^ crossing beyond the threshold for exocytosis (*top row*, *red dashed line*), synaptic plasticity could be progressively changed to its opposite depending on the type of gliotransmitter. Astrocytic glutamate could thus turn facilitating synapses into depressing (*middle row*) whereas astrocyte-derived ATP or adenosine could turn depressing synapses into facilitating (*bottom row*). Simulations are based on a model of astrocyte-regulation of synaptic release introduced in De Pittà et al. (2011). “Basal Synaptic Release Probability” in **(B)** refers to the probability of synaptic release at rest, that is when synaptic activity is assumed to be very low and the amount of neurotransmitter released upon arrival of an action potential to the presynaptic terminal is essentially independent of previous release events (Zucker and Regehr, 2002; De Pittà et al., 2011).

Astrocyte-derived glutamate and ATP or adenosine can modulate synaptic transmission, either increasing neurotransmitter release (Araque et al., 1998a,b; Fiacco and McCarthy, 2004; Jourdain et al., 2007; Perea and Araque, 2007; Bonansco et al., 2011; Di Castro et al., 2011; Panatier et al., 2011) or decreasing it (Zhang et al., 2003, 2004a; Pascual et al., 2005; Andersson and Hanse, 2010, 2011) depending on the type of presynaptic receptor involved and the brain area (pathway **B** in Figure 1B; see also Table 1). At excitatory synapses in the hippocampal dentate gyrus, glutamate is released from surrounding astrocytic processes in close proximity to presynaptic NR_2_B-containing NMDA receptors (Figure 4A). Activation of these receptors results in increased synaptic release and strengthening of synaptic transmission (Jourdain et al., 2007). At Schaffer collateral synapses in the CA1 hippocampal area a similar effect is mediated by presynaptic mGluRs (Fiacco and McCarthy, 2004; Perea and Araque, 2007). Besides directly targeting presynaptic receptors, astrocyte-released glutamate could also bind ionotropic receptors found along the axons of hippocampal CA3 pyramidal cells, broadening the profile of propagating action potentials (APs) (Sasaki et al., 2011). The broadened APs in turn, trigger larger Ca^2+^ elevations in presynaptic boutons, resulting in larger synaptic release probability.

Conversely, astrocyte-released ATP and its derivative adenosine, bind to presynaptic PRs, i.e. P_2_Y_1_Rs or A_1_Rs, respectively, decreasing synaptic release (Zhang et al., 2003, 2004a; Pascual et al., 2005). However, astrocyte-derived adenosine, could also target A_2A_ receptors which can increase synaptic release (Panatier et al., 2011) (omitted from Figures 1A and 4A for simplicity). Both effects of adenosine—inhibitory via A_1_Rs and stimulatory via A_2A_Rs—have been described at hippocampal CA3-CA1 synapses (Zhang et al., 2003, 2004a; Pascual et al., 2005; Serrano et al., 2006; Panatier et al., 2011), and the prevalence of one on the other likely depends on the level of synaptic activity (Panatier et al., 2011). This would be in line with observations at the frog's neuromuscular junction, where different stimulations of the tibial nerve differentially activated A_1_Rs or A_2A_Rs in association with different Ca^2+^ dynamics in the peri-junctional glial cell (Todd et al., 2010). Intriguingly, at hippocampal synapses different synaptic stimuli could differentially change the morphology of astrocyte perisynaptic processes (Haber et al., 2006; Lavialle et al., 2011) thus reshaping, in an activity-dependent fashion, the extracellular space of interaction between astrocyte-derived adenosine and presynaptic receptors. This could dynamically regulate access of adenosine to one receptor with respect to the other (Haber et al., 2006; Syková and Nicholson, 2008) ultimately modulating synaptic release in a complex fashion. The functional consequences of astrocytic remodeling were indeed demonstrated in the supraoptic nucleus, where dynamic changes in the astrocytic wrapping of synapses during lactation could regulate the extent of synaptic glutamate spillover and thereby control heterosynaptic depression of GABAergic transmission by presynaptic mGluRs (Oliet et al., 2001; Piet et al., 2004).

The opposite effects due to astrocytic glutamate or ATP/adenosine could endow astrocytes with the capacity to exert non-stereotyped bimodal control of synaptic transmission (Volterra and Meldolesi, 2005). On the other hand, the temporal concurrence of both these effects due to co-expression of inhibitory and stimulatory receptors at the same synaptic terminals (Shigemoto et al., 1997; Rebola et al., 2005), could result in occlusion, i.e. no net effect on synaptic release by the astrocyte (De Pittà et al., 2011). Alternatively, balanced activation (and possibly occlusion) of A_1_ and A_2A_ receptors by astrocyte-derived adenosine could set synaptic release in basal conditions (Panatier et al., 2011). Hence, the ensuing regulation of synaptic transmission triggered by gliotransmitters in response to stimuli, could result instead from an unbalance of activation of these receptors rather than by the distinct activation of one receptor type with respect to the other, ultimately providing a high degree of complexity in the control of synaptic transmission by astrocytes. For the sake of clarity, in the following we will consider only release-decreasing effects of astrocyte ATP or its derivative, adenosine.

The details of the biochemical mechanism underlying modulation of synaptic release by astrocytic glutamate or ATP (or adenosine) likely depend on the type of targeted presynaptic receptors and are not fully understood (Pinheiro and Mulle, 2008). The simplest explanation would be that astrocytic glutamate and ATP lead to a modulation of presynaptic intracellular Ca^2+^ levels which eventually results in a modulation of synaptic release probability (Zucker and Regehr, 2002; Pinheiro and Mulle, 2008) with significant repercussions on synaptic plasticity, including short-term depression and facilitation.

Short-term facilitation and depression can be assessed by measuring the paired-pulse ratio (PPR), i.e. the ratio between the amplitudes of successive postsynaptic currents (PSCs) recorded in response to a pair of action potentials in rapid succession as illustrated in Figure 4B (Zucker and Regehr, 2002). When the value of the peak postsynaptic current associated to the second incoming spike is larger than the peak current recorded in coincidence with the first spike (*green traces*), then synaptic release is increasing for incoming spikes, i.e. facilitation occurs, and the corresponding PPR is larger than unity. Conversely, when the second peak is less than the first peak (*magenta traces*), this marks a decrease of neurotransmitter release from the presynaptic terminal which reflects depression, and the corresponding value of PPR is less than one. It should be noted that, by varying the interval between two pulses, the same synapse can be either depressed or facilitated. For simplicity we omit the interpulse interval as a variable in this description.

In general, in basal conditions, i.e. in response to an individual action potential, the value of synaptic release probability of an individual synapse or of an ensemble of synapses defines the nature of synaptic transmission at that synapse/ensemble, namely whether it is facilitating or depressing, with low values of probability favoring facilitation and high values favoring depression (Abbott and Regehr, 2004) (Figure 4B, *right*). Thus, any modulation of synaptic release probability by gliotransmitters that changes the PPR from below unity to values above it or vice versa, could switch the mode of synaptic transmission from depressing to facilitating or vice versa. This scenario was theoretically addressed in De Pittà et al. (2011) and it was shown to substantially agree with experiments. Indeed, at hippocampal synapses, the increase of synaptic release probability due to astrocytic glutamate correlates with a *decrease* of the PPR (Jourdain et al., 2007; Perea and Araque, 2007; Bonansco et al., 2011). Conversely, a decrease of synaptic release due to the action of astrocyte-derived ATP (or adenosine) is accompanied by an *increase* of the PPR (Zhang et al., 2003, 2004a). The frequency of astrocytic glutamate (Glu) and/or ATP exocytosis are likely crucial in the regulation of the extent of the modulation of synaptic release by astrocytes (De Pittà et al., 2011), a principle illustrated in Figure 4C. This figure shows the simulated peak postsynaptic currents for the same synapse in response to 100 trials of presynaptic spike trains with identical statistics (*raster plots*). Each column in Figure 4C corresponds to a different frequency of astrocytic Ca^2+^ pulses (*top row*), yielding to different frequencies of gliotransmitter release when Ca^2+^ crosses the threshold for exocytosis (*dashed red line*) (see also Figure 3B). Colors in the raster plots refer to paired-pulse plasticity quantified by PPR. For two consecutive presynaptic spikes, if the second spike releases synaptic neurotransmitter more than the first spike, then it is PPR > 1, paired-pulse facilitation is observed, and the peak postsynaptic current associated to the second spike is colored in *green*. Vice versa, if the amount of neurotransmitter released by the second spike is less than that released by the first one, then PPR < 1, that is paired-pulse depression occurs, and the second peak postsynaptic current is colored in *magenta*.

In absence of astrocytic gliotransmitter (*left column*), the raster plot of a facilitating synapse (*middle row*) shows predominant paired-pulse facilitation, i.e. mostly *green* dots. However, in presence of release of glutamate from the astrocyte (+Glu), as explained above, the stimulatory effect of this gliotransmitter on synaptic release changes paired-pulse plasticity which is marked by the appearance of *magenta* bands in the raster plots (*middle* and *right columns*). These bands locate time intervals where paired-pulse depression becomes prominent (i.e. predominance of *magenta* dots) on the overall paired-pulse facilitation background (*green dots*). Notably these bands are almost in coincidence with glutamate release from the astrocyte (*top row*) and their number increases with the glutamate release frequency. Therefore, for the same time window, the same originally-facilitating synapse gets increasingly depressing as the rate of glutamate release from the astrocyte increases. The opposite is observed for an otherwise depressing synapse (i.e. predominance of paired-pulse depression, thus mostly *magenta* dots in the *bottom left* raster plot). In this case, in presence of release of ATP and its derivative adenosine (+ATP/Adn) from the astrocyte, due to the inhibitory effect of astrocyte-derived purines on synaptic release considered in this example, *green* bands appear in the raster plots which mark the onset of periods of predominant paired-pulse facilitation. The number of these bands grows for higher rates of ATP release from the astrocyte so that the same originally-depressing synapse behaves more akin of a facilitating one as the rate of ATP release from the astrocyte increases.

**Figure 5:**
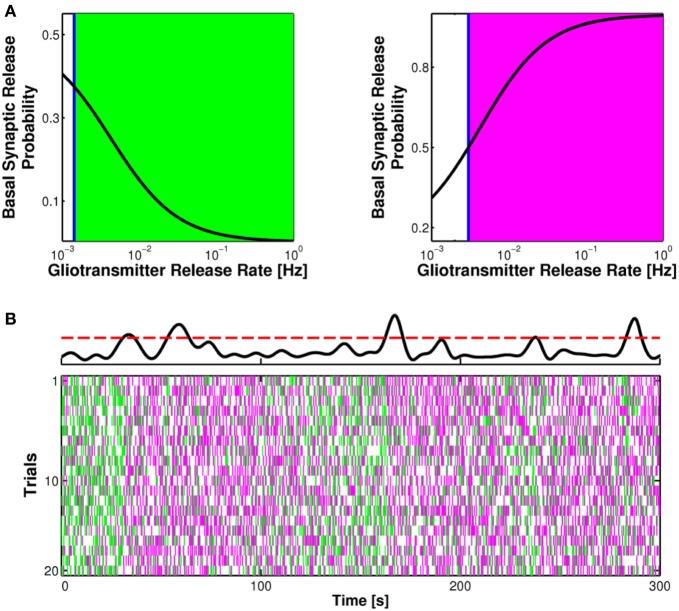
**Short-term synaptic plasticity is physiologically set by the rate of gliotransmitter release from the astrocyte. (A)** The rate of glutamate or ATP release from the astrocyte could differently affect basal synaptic release probability. In particular, a threshold frequency for the release of these gliotransmitters could exist (*blue vertical line*) beyond which a depressing synapse could turn into facilitating (*right*, *green area*) or vice versa, a facilitating synapse could become depressing (*left*, *magenta area*). Adapted from De Pittà et al. (2011). **(B)** In basal conditions, synaptic release is due to sporadic neuronal network firing and the possible frequency of Ca^2+^ fluctuations beyond the threshold for exocytosis (*dashed red line*) in the astrocyte (*top trace*). In this fashion plastic changes in paired-pulse ratio could be inherently regulated by astrocytic gliotransmitters, as shown here for the case of an originally facilitating synapse under the effect of astrocytic glutamate exocytosis (20 trials with identical input statistics). Adapted from Berry et al. (2011). The color code for the raster plot is the same as in Figure 4C.

An intriguing prediction that follows from the above arguments is that the frequency of gliotransmitter release, by modulating synaptic release probability at basal conditions, could dynamically control the nature of synaptic transmission as elucidated in Figure 5A. In particular, under certain conditions, a threshold frequency for gliotransmitter release (*blue line*) could exist above which the astrocyte can switch the nature of synaptic transmission, turning depressing synapses into facilitating or vice versa, facilitating synapses into depressing (De Pittà et al., 2011). Hence, the plasticity mode at such synapses is not fixed but rather is set by the release rate of gliotransmitters from neighboring astrocytic processes.

Remarkably, the threshold frequency of gliotransmitter release that discriminates between facilitation and depression at one synapse can be as low as 0.05 Hz or less (as in the case of Figure 5A) thus falling within the range of Ca^2+^ oscillations observed in astrocytes in basal conditions (Parri et al., 2001; Aguado et al., 2002; Bonansco et al., 2011; Di Castro et al., 2011). In such conditions as shown in Figure 5B (*top*), intracellular Ca^2+^ levels in astrocytes spontaneously fluctuate in a highly stochastic fashion (Hirase et al., 2004; Di Castro et al., 2011) and can similarly cross the threshold for gliotransmitter release. The ensuing release of gliotransmitter, however, could be sufficient to tonically set the basal tone of synaptic transmission (Figure 5B). Inasmuch as the rate of gliotransmitter release could also correlate with the incoming synaptic stimulus through Ca^2+^ dynamics in the astrocytes (Aguado et al., 2002; Di Castro et al., 2011), this scenario discloses the possibility that astrocytes act as endogenous regulators of the efficacy of synaptic transmission (Haydon, 2001; Giaume et al., 2010; Halassa and Haydon, 2010; Di Castro et al., 2011; Panatier et al., 2011). That is, through integration of synaptic activity by means of their Ca^2+^ signals, astrocytes could adapt synaptic strength according to the history of the synapse.

## Implications of astrocyte modulation of synaptic transmission and plasticity

The effect of modulation of synaptic release probability by astrocytic gliotransmitters may decay more slowly than the Ca^2+^ elevation triggering astrocyte exocytosis (Fiacco and McCarthy, 2004; Serrano et al., 2006), and still be present upon gliotransmitter release by following Ca^2+^ increases (Volman et al., 2007). Therefore astrocytic Ca^2+^ activity resulting in high rates of gliotransmitter release, could bring forth long-lasting modulations of synaptic release. Insofar as synaptically-released neurotransmitter could shape postsynaptic signaling at the basis of long-term synaptic plasticity (Bliss and Collingridge, 1993), persistent modulations of synaptic release probability by astrocyte could ultimately contribute to long-term changes of synaptic strength underlying neural processing, memory formation and storage of information. Support to this scenario comes from studies on hippocampal synapses for which the temporal coincidence of postsynaptic depolarization with the increase of Ca^2+^ elevations in neighboring astrocytes was shown to induce long-term potentiation (LTP) of synaptic transmission (Perea and Araque, 2007; Navarrete and Araque, 2010; Navarrete et al., 2012a,b). Notably, this form of LTP is independent of the activation of postsynaptic NMDARs but rather, it depends on glutamate released from astrocytes, which persistently potentiates synaptic transmitter release through activation of presynaptic mGluRs (Perea and Araque, 2007). On the other hand, at synapses between excitatory neurons in layers 4 and 2/3 in the rat barrel cortex, activity-dependent induction of long-term depression (LTD) also requires astrocyte Ca^2+^ signaling (Min and Nevian, 2012). At these synapses, postsynaptically-released endocannabinoids mediate Ca^2+^-dependent release of glutamate from astrocytes which targets presynaptic NMDARs bringing forth LTD (Sjöström et al., 2003; Rodríguez-Moreno and Paulsen, 2008).

The differential induction of LTP and LTD in neighboring synapses has been suggested to determine the size and shape of cortical functional topographic units such as ocular dominance columns in the primary visual cortex and whisker barrels in the primary somatosensory cortex (Feldman and Brecht, 2005; Hensch, 2005; Li et al., 2009). Thus, the possible involvement of astrocytes in LTP and LTD suggests that these cells could contribute to the plasticity of cortical maps and the development of corresponding sensory representations (Rossi, 2012).

Modulation of synaptic release probability by astrocytic gliotransmitters could also alter the temporal order of correlated pre- and postsynaptic spiking that critically dictates spike-timing-dependent plasticity (STDP) (Pascual et al., 2005). Inhibition of spontaneous glutamate release from astrocytes in hippocampal CA1 synapses, consistent with a decrease of synaptic release probability, was indeed reported to modify the threshold for induction of spike-timing-dependent LTP (Bonansco et al., 2011). In this fashion, astrocytes by gliotransmission-mediated regulations of synaptic release probability could control not only different mechanisms of synaptic plasticity but also the threshold of synaptic activity required for their onset, thus playing a role in metaplasticity too, that is the plasticity of synaptic plasticity (Abraham, 2008).

Gliotransmitters do not control synaptic plasticity only via presynaptic actions, but also by actions on postsynaptic receptors. Indeed, the induction of LTP itself appears to be uniquely controlled by astrocytes through the release of D-serine (Santello and Volterra, 2010) (pathway **C** in Figure 1B). Both at hippocampal and cortical synapses astrocytic D-serine rather than glycine, is the endogenous co-agonist of synaptic NMDA receptors (Henneberger et al., 2010; Takata et al., 2011; Papouin et al., 2012). By controlling the level of co-agonist site occupancy of postsynaptic NMDARs, astrocytic D-serine affects the level of activation of these receptors and thus activity-dependent long-term synaptic changes (Bains and Oliet, 2007). In particular, the additional burst of activation of postsynaptic NMDARs (about 25% more) induced by astrocyte D-serine release seems necessary for LTP induction (Henneberger et al., 2010). Moreover, in conditions of reduced synaptic coverage by astrocytes, such as during lactation in the hypothalamus, higher presynaptic activity is required to obtain LTP while the same level of activity that normally induces LTP results instead in LTD (Panatier et al., 2006). In such conditions, astrocyte-released D-serine is diluted in the larger extracellular space resulting in a reduced number of postsynaptic NMDARs recruited by synaptic activity, which ultimately translates into smaller postsynaptic Ca^2+^ increases. Therefore, experimental protocols that would be expected to cause LTP, elicit LTD instead. This is in agreement with the Bienenstock-Cooper-Munro (BCM) model for variation of the threshold for LTP, which predicts that the relationship between synaptic activity and persistent changes in synaptic strength can vary according to the number of NMDARs available during synaptic activation (Bienenstock et al., 1982; Abraham and Bear, 1996). Effectively, by adjusting the D-serine occupancy of the NMDAR co-agonist-binding site, astrocytes can shift the relationship between activity and synaptic strength (Panatier et al., 2006).

Besides D-serine, experimental evidence hints that glutamate and ATP released from astrocytes could also directly bind postsynaptically-located receptors and accordingly, play a role in regulation of long-term synaptic plasticity. In the paraventricular nucleus of the hypothalamus for example, ATP released from astrocytes could directly target postsynaptic P_2_X_7_ receptors, promoting insertion of postsynaptic AMPARs which results in LTP of synaptic transmission (Gordon et al., 2005). Interestingly, ATP release is mediated by Ca^2+^ dynamics triggered in astrocytes by noradrenergic afferents which, in the hypothalamus, generally lack direct postsynaptic contacts (Sawyer and Clifton, 1980), thus hinting that signaling in this vital homeostatic circuit may require dynamic neuron-glia interactions.

Glutamate released from astrocyte could also target extrasynaptically-located NR_2_B-containing NMDA receptors at postsynaptic terminals, triggering slow inward currents (SICs) (Fellin et al., 2004; D'Ascenzo et al., 2007; Navarrete and Araque, 2008; Bardoni et al., 2010; Pirttimaki et al., 2011; Navarrete et al., 2012a,b) mainly mediated by Ca^2+^ ions (Cull-Candy et al., 2001), whose depolarizing action could affect postsynaptic neuronal firing (D'Ascenzo et al., 2007; Pirttimaki et al., 2011). In the primary visual cortex, nucleus basalis-mediated cholinergic activation of astrocytes mediates an increase of SICs frequency which correlates with a long-lasting increase of firing activity in visual responses of V1 excitatory neurons (Chen et al., 2012). The ensuing modulations of firing activity of these neurons by astrocyte-mediated SICs might ultimately affect STDP at individual synapses controlling orientation-specific responses of V1 neurons to visual stimuli (Jia et al., 2010).

Modulation of synaptic transmission by Ca^2+^-dependent gliotransmission may not be limited to the very synapses that trigger Ca^2+^ activity in the astrocyte but it could also affect farther synaptic domains in a multimodal fashion (Kozlov et al., 2006), depending both on the morphology of the sites of astrocyte-synapse reciprocal communication (Ventura and Harris, 1999; Haber et al., 2006) and the functional connectivity of the astrocytic network (Pannasch et al., 2011). High-frequency activity of a Schaffer collateral fiber can trigger the potentiation of synaptic transmission at the same fiber but heterosynaptic suppression of another, adjacent fiber, by inducing ATP release from an astrocyte interposed between the two fibers (Zhang et al., 2003, 2004a; Pascual et al., 2005). In the somatosensory cortex in particular, astrocyte-mediated heterosynaptic suppression could modulate GABAergic inhibitory transmission (Benedetti et al., 2011) which plays a dominant role in the control of cortical neuronal excitability (Swadlow, 2002). Given that both experimental observations and theoretical arguments suggest that excitation and inhibition are globally balanced in cortical circuits (Shadlen and Newsome, 1994; Troyer and Miller, 1997; Shu et al., 2003; Haider et al., 2006), one may speculate that this mechanism could be involved in gating of signal transmission (Buzsáki, 2010). That is, by modulating inhibitory synaptic transmission, astrocytes could favor network excitation resulting in neuronal firing consistent with the transmission, i.e. gating “on,” of specific stimuli rather than others (Vogels and Abbott, 2009).

The latter idea could also bring to a possible role of astrocyte signaling in coherent function of neural networks underlying potential behavioral states (Engel et al., 2001). In cortical slices, for example, stimulation of a single astrocyte was reported to activate large portions of the astrocytic network and to result in an increase of synchronized neuronal depolarizations (Poskanzer and Yuste, 2011). This phenomenon was suggested to modulate the induction of cortical UP and DOWN states, possibly involved in determining the oscillatory activity observed in slow-wave sleep (Fellin et al., 2004; Halassa and Haydon, 2010; Poskanzer and Yuste, 2011), and is consistent with reports of sleep perturbations in mice lacking astrocytic gliotransmitter exocytosis (Fellin et al., 2009; Halassa et al., 2009; Fellin et al., 2012).

## Conclusions

A large body of evidence has accumulated over the last years, suggesting an active role of astrocytes in many brain functions. Collectively these data have fuelled the concept that synapses could be tripartite rather than bipartite, since in addition to the pre- and post-synaptic terminals, the astrocyte could be an active element in synaptic transmission (Araque et al., 1999; Haydon, 2001). While the tripartite synapse concept captures well the essence of astrocyte-regulated synapses, the inclusion of astrocytic signaling within our current knowledge of synaptic transmission could add more than just one level of complexity. Existing evidence suggests that astrocytes could produce not just tonic and diffuse modulatory influences on synapses but also engage in more focused, spatially precise and constrained communications with synaptic terminals (Anderson and Swanson, 2000; Jourdain et al., 2007; Santello and Volterra, 2009; Bergersen et al., 2012). This calls to rethink the definition of a functional synapse, to include the contribution from surrounding astrocytes. To conclude, the growing appreciation that astrocytes can regulate synaptic information at many levels, from activity of single synapses to network levels and behavioral states (Fellin et al., 2009; Halassa et al., 2009; Zorec et al., 2012) changes our understanding of brain communication and the role of glial cells in synaptic transmission. This resulting novel scenario offers an enticing platform for future theoretical investigations that we are just beginning to appreciate in its potential far-reaching implications.

### Conflict of interest statement

The authors declare that the research was conducted in the absence of any commercial or financial relationships that could be construed as a potential conflict of interest.

## Abbreviations

Adn: adenosine
AFM: amplitude and frequency modulation
AM: amplitude modulation
AMPA: 2-amino-3-(5-methyl-3-oxo-1,2- oxazol-4-yl) propanoic acid
AMPAR: AMPA glutamate receptor
AP: action potential
ATP: adenosine triphosphate
Ca^2+^: calcium
CICR: Ca^2+^-induced Ca^2+^ release
D-ser: D-serine
EAAT: excitatory aminoacid transporter
ER: endoplasmic reticulum
FM: frequency modulation
GABA: γ-aminobutyric acid
GJC: gap-junction channel
Glu: glutamate
GluR: glutamate receptor
mGluRs: metabotropic glutamate receptor
GPCR: G_q_ protein-coupled receptor
IP-5P: inositol polyphosphate 5-phosphatase
IP_3_: inositol 1,4,5-trisphosphate
IP_3_-3K: IP_3_ 3-kinase
IP_3_R: IP_3_ receptor
LTD (LTP): long-term depression (potentiation)
NMDA: *N*-methyl-d-aspartic acid
NMDAR: NMDA glutamate receptor
PLCδ (PLCβ): phospholipase Cδ (Cβ)
PPR: paired-pulse ratio
PR: purinergic receptor
PSC: postsynaptic current
SIC: slow inward current
SNARE: soluble N-ethylmaleimide-sensitive factor attachment protein receptor
STDP: spike-timing-dependent plasticity
TACE: TNFα-converting enzyme
TNFα: tumor necrosis factor-α.
